# Computed tomography versus plain radiography assessment of acetabular fracture reduction is more predictive for native hip survivorship

**DOI:** 10.1007/s00402-019-03192-w

**Published:** 2019-04-27

**Authors:** Diederik O. Verbeek, Jelle P. van der List, David L. Helfet

**Affiliations:** 1 Orthopaedic Trauma Service, Hospital for Special Surgery and New York Presbyterian Hospital, Weill Cornell Medicine, New York, NY USA; 2 Present Address: Trauma Research Unit, Department of Surgery, Erasmus MC, University Medical Center Rotterdam, Rotterdam, The Netherlands

**Keywords:** Acetabular fracture, Hip survivorship, Total hip arthroplasty, Pelvic radiography, Computed tomography, Postoperative reduction

## Abstract

**Introduction:**

Computed tomography (CT) is more accurate than plain pelvic radiography (PXR) for evaluating acetabular fracture reduction. As yet unknown is whether CT-based assessment is more predictive for clinical outcome. We determined the independent association between reduction quality according to both methods and native hip survivorship following acetabular fracture fixation.

**Materials and methods:**

Retrospectively, 220 acetabular fracture patients were reviewed. Reductions on PXR were graded as adequate or inadequate (0–1 mm or > 1 mm displacement) (Matta’s criteria). For CT-based assessment, adequate reductions were defined as < 1 mm step and < 5 mm gap, and inadequate reductions as ≥ 1 mm step and/or ≥ 5 mm gap displacement. Predictive values and Kaplan–Meier hip survivorship curves were compared and risk factors for conversion to total hip arthroplasty (THA) were identified.

**Results:**

Mean follow-up was 8.9 years (SD 5.6, range 0.5–23.3 years), and 52 patients converted to THA (24%). Adequate reductions according to CT versus PXR assessment were associated with higher predictive values for native hip survivorship (92% vs. 82%; *p* = 0.043). Inadequate reductions were equally predictive for conversion to THA (33% for CT and 30% for PXR; *p* = 0.623). For both methods, survivorship curves of adequate versus inadequate reductions were significantly different (*p* = 0.030 for PXR, *p* < 0.001 for CT). Only age ≥ 50 years (*p* < 0.001) and inadequate reductions as assessed on CT (*p* = 0.038) were found to be independent risk factors for conversion to THA. Reduction quality as assessed on PXR was not found to be independently predictive for this outcome (*p* = 0.585).

**Conclusion:**

Native hip survivorship is better predicted based on postoperative CT imaging as compared to PXR assessment. Predicting need for THA in patients with inadequate reductions based on both assessment methods remains challenging. While both PXR and CT-based methods are associated with hip survivorship, only an inadequate reduction according to CT assessment was an independent risk factor for conversion to THA.

## Introduction

Native hip survivorship in patients who receive operative treatment for acetabular fractures is influenced by a variety of factors [1–3]. These include non-modifiable or predetermined factors, such as patient age, fracture impaction, posterior wall involvement, and damage to the femoral head. An important modifiable risk factor thought to be predictive for clinical outcomes is quality of acetabular fracture reduction [3, 4–7]. An anatomic postoperative reduction has widely been used as a (early) proxy for successful surgical treatment.

Yet, a number of studies have noted that up to one-third of patients with an apparent anatomic reduction have poor clinical outcome following acetabular fracture surgery [6–8]. This seemingly contradictory finding may in part be attributed to the specific method used to assess reduction quality. In general, Matta’s criteria based on plain pelvic radiography (PXR) are used for this purpose (Matta). However, it is currently well established that CT is superior in detecting residual fracture displacement and different criteria to grade accuracy of reduction may have to be applied for this modality [9–12].

While it is evident that CT is more accurate than PXR for evaluation of acetabular fracture reduction, it remains to be determined whether CT-based assessment using criteria specifically designed for this modality is also more predictive for clinical outcome. Therefore, the purpose of this study is to determine the (independent) association between reduction quality according to both methods and native hip survivorship following acetabular fracture fixation.

## Materials and methods

### Study design

Following Institutional Review Board approval, a search in our Orthopedic Trauma Service (OTS) registry was performed for all adult patients who underwent surgical fixation for an acute (within 3 weeks of injury) isolated acetabular fracture between the dates of January 1994 and June 2014. Surgical indications for open reduction and internal fixation (ORIF) of acetabular fractures were ≥ 2 mm fracture displacement within the weight-bearing dome, an incongruent hip joint, or an unstable posterior wall fracture. All surgeries were performed by the senior author. Within the first postoperative week, all patients routinely underwent PXR in three standard radiographic views (antero-posterior, iliac oblique, and obturator oblique) as well as pelvic CT imaging.

### Inclusion and exclusion criteria

A total of 481 consecutive acetabular fracture patients were found to be eligible for inclusion. Patients were excluded if follow-up was less than 2 years; unless they converted to total hip arthroplasty (THA) in this interval (208 exclusions), if they had concomitant femoral head fractures (six exclusions) or pelvic ring fractures (two exclusions), or if postoperative PXR and/or CT imaging was not available for review (45 exclusions). This left 220 patients to be included in the study.

### Data collection

Data were retrospectively collected from our prospective OTS registry and included patient demographics and fracture types, as classified by the senior author (***) using the Letournel acetabular fracture classification system [8, 11]. Patients were routinely followed up in clinic at regular intervals (at 2 weeks and 1, 3, 6, and 12 months, and annually thereafter). In addition, all patients were contacted by mail and telephone to determine the most current status of their native hip (preserved native hip versus failed native hip with conversion to THA).

### Quality of reduction

Postoperative PXRs were assessed for residual gap and/or step displacement in the three standard views and the greatest measurement was used to grade quality of reduction according to Matta’s system [6]. In accordance with prior studies, adequate (or anatomic) reductions with 0–1 mm of displacement were compared to inadequate (imperfect or poor) reductions with > 1 mm displacement [2, 3]. For the CT-based method, postoperative pre-digital (prior to 2000) and digital CT images were independently assessed in the axial, sagittal, and coronal planes, and residual gap and step displacement were measured along the articular surface at the level of the weight-bearing dome. Adequate reductions on postoperative CT were defined as < 1 mm step and < 5 mm gap displacement and inadequate reductions as ≥ 1 mm step and/or ≥ 5 mm gap displacement [12]. Assessment of postoperative PXR’s and CT scans was performed by two observers (**, **) and differences settled in consensus. The observers were blinded for clinical outcome and not involved in the initial surgical care of the included patients.

### Statistical analysis

Statistical analysis was performed using SPSS version 24.0 (IBM Software, Armonk, NY, USA). Baseline characteristics of continuous variables were presented as means with standard deviation (SD) and ranges, and nominal data as number (*n*) with percentage (%). Two-by-two tables were constructed to show the relation between quality of reduction (adequate versus inadequate) as assessed according to the PXR and CT-based methods and native hip survivorship. Predictive values for both methods were compared using Pearson’s Chi-square or Fisher’s exact tests when appropriate. Kaplan–Meier curves were used to plot native hip survivorship for reduction quality based on both PXR and CT assessment, and log-rank tests were used to determine statistical differences. Cox regression analysis was used to identify dependent and independent risk factors associated with conversion to THA. Results are presented as hazard ratio’s (HR) with 95% confidence intervals (95% CI). A *p* value of < 0.05 was considered to indicate a statistically significant result.

## Results

### Baseline characteristics

A total of 220 patients were included in this study. Mean age was 50.8 years (SD 17.8, range 18–91 years); 117 patients (53%) were ≥ 50 years of age and 142 patients (64%) were male. The majority of patients had associated type fractures (64%); (Table 1). Posterior wall impaction was found in 77 patients (18%) and supero-medial dome impaction in 40 patients (9%).

**Table 1:** Incidence of different fracture types in the total study cohort (*n* = 220)

Acetabular fracture type	
Elementary	
Anterior column	12 (6%)
Anterior wall	3 (1%)
Posterior column	0 (0%)
Posterior wall	48 (22%)
Transverse	16 (7%)
Associated	
T-shaped	8 (4%)
Transverse with posterior wall	42 (19%)
Posterior column with posterior wall	5 (2%)
Anterior column with posterior hemitransverse	46 (21%)
Both column	40 (18%)

Mean follow-up was 8.9 years (SD 5.6, range 0.5–23.3 years). A total of 52 patients had native hip failure and converted to THA (24%) at a mean of 4.2 years (SD 4.2 range 0.3–21.0 year); 27 patients (52%) had early failure (within 2 years). Indication for conversion to THA was symptomatic osteoarthritis in 43 patients (83%). A further five patients (10%) had avascular necrosis of the femoral head, 3 (6%) had fixation failure, and 1 (2%) a deep infection; all of these patients had poor reductions on both PXR and CT.

### Quality of reduction

An adequate reduction according to CT assessment (as compared to PXR) was associated with a higher predictive value for native hip survivorship (92% versus 82%; *p* = 0.043); (Tables 2, 3). Inadequate reductions according to both methods were equally predictive for conversion to THA (33% for CT and 30% for PXR; *p* = 0.623). Of 118 adequate PXR reductions, 61 (52%) were graded inadequate on CT. Of these “missed” incongruencies 16 (26%) converted to THA.

**Table 2:** Quality of acetabular reduction, according to pelvic radiography (PXR) assessment versus native hip survivorship in the total cohort (*n* = 220)

Total cohort	Hip survivorship		
	Yes	No	Total
Reduction on PXR			
Adequate	97 (82%)	21 (18%)	118 (100%)
Inadequate	71 (70%)	31 (30%)	102 (100%)
Total	168 (76%)	52 (24%)	220 (100%)

**Table 3:** Quality of acetabular reduction, according to CT-based assessment versus native hip survivorship in the total cohort (*n* = 220)

Total cohort	Hip survivorship		
	Yes	No	Total
Reduction on CT			
Adequate	77 (92%)	7 (8%)	84 (100%)
Inadequate	91 (67%)	45 (33%)	136 (100%)
Total	168 (76%)	52 (24%)	220 (100%)

### Native hip survivorship

**Fig. 1:**
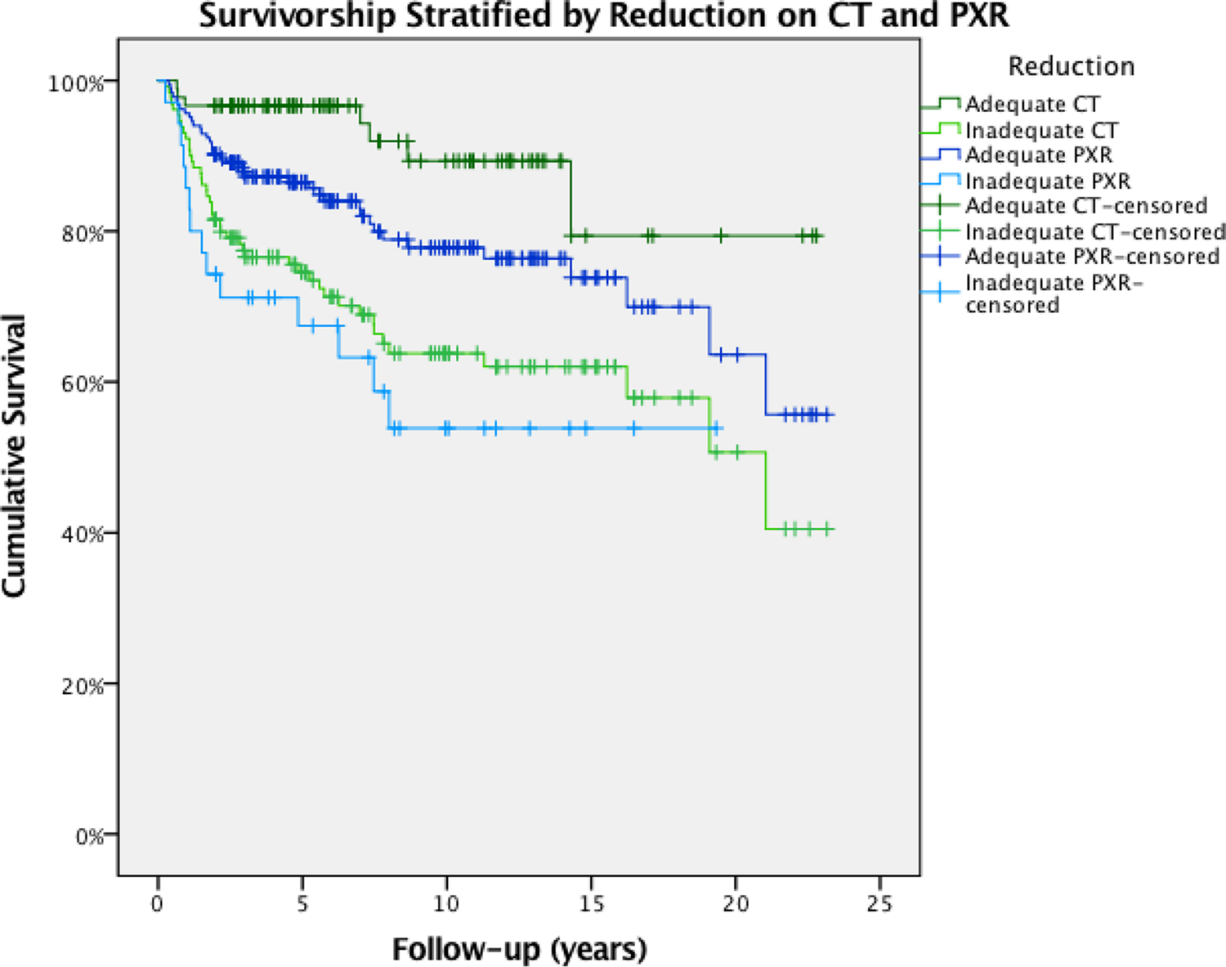
Native hip survivorship curves for reduction quality based on both PXR and CT assessment

Hip survivorship curves for reduction quality based on both PXR and CT assessment are shown in Fig. 1. For both methods, curves of adequate versus inadequate reductions were significantly different according to log-rank tests (*p* = 0.030 for PXR and *p* < 0.001 for CT). Ten-year native hip survivorship was higher for patients with an adequate versus inadequate reduction according to PXR assessment (79%; 95% CI 71–88%) versus 68% (95% CI 56–77%) as well as based on CT assessment (89%; 95% CI 81–98%) versus 65% (95% CI 55–73%).

Cox regression analysis identified the following (dependent) risk factors for conversion to THA; age ≥ 50 years (*p* < 0.001), posterior wall (*p* = 0.002), and supero-medial dome impaction (*p* < 0.001) and inadequate reductions as assessed on PXR (*p* = 0.019) and CT (*p* < 0.001) (Table 4). After adjusting for confounders, only age ≥ 50 years (*p* < 0.001) and an inadequate reduction quality as assessed on CT (*p* = 0.038) were found to be (independent) risk factors. Reduction quality as assessed on PXR was not found to be independently predictive for native hip failure (*p* = 0.585).

**Table 4:** Cox regression analysis for (dependent and independent) risk factors associated with conversion to total hip arthroplasty in all patients (*n* = 220)

	Unadjusted		Adjusted	
	HR (95% CI)	*p* value	HR (95% CI)	*p* value
Age (≥ 50 years)	5.3 (2.6–11.1)	< 0.001	4.6 (2.2–9.6)	< 0.001
Gender (female)	1.5 (0.9–2.6)	0.140	1.5 (0.9–2.7)	0.129
Fracture type (Associated)	1.5 (0.8–2.8)	0.166	0.9 (0.5–1.7)	0.730
Posterior wall impaction	2.4 (1.4–4.1)	0.002	1.7 (0.9–3.0)	0.081
Supero-medial dome impaction	2.9 (1.7–5.2)	< 0.001	1.7 (0.9–3.2)	0.136
Reduction on PXR (inadequate)	1.8 (1.1–3.2)	0.033	1.2 (0.6–2.3)	0.585
Reduction on CT (inadequate)	4.1 (1.9–9.2)	< 0.001	2.6 (1.1–6.2)	0.038

*PXR* plain pelvic radiography

## Discussion

Results of this study indicate that following ORIF of acetabular fractures, an adequate reduction according to CT-based assessment is more predictive for native hip survivorship than PXR-based assessment.

The improved performance of CT in this regard is likely due to its ability to more accurately detect even minor incongruencies within the acetabular joint surface. The previous studies have shown definitively that both pre- and post-operative CT is a superior modality for evaluation of acetabular fracture displacement [9, 10, 11,13]. The current study does not attempt to replicate these findings, but logically follows earlier work. In a prior study, PXR versus CT assessment of postoperative acetabular reduction was compared using the Matta criteria [11]. Results showed that 75% of anatomic reductions (0–1 mm displacement) on PXR were classified imperfect or poor (≥ 2 mm displacement) on CT. The question whether superior accuracy would result in better performance for CT in terms of predicting hip survivorship remained unanswered as it could be argued that more clinically irrelevant displacements may be detected using this modality. Based on our current results, it appears that at least some malreductions, which go undetected on PXR, can lead to symptomatic osteoarthritis ultimately necessitating THA conversion. These “missed” incongruencies may be of importance at long-term follow-up particularly in more active younger adults.

In the current study, we chose to grade postoperative reductions on CT based on a system specifically designed for this modality (as the commonly used Matta criteria for reduction quality on PXR have not been validated for post-operative CT assessment [12]. For analysis of PXR results, adequate (or anatomic) reductions were compared to inadequate (or non-anatomic) reductions, similar to earlier studies [2, 3].

Our results further show that contrary to prediction of hip survivorship, prediction of hip failure based primarily on reduction quality (on PXR or CT) remains problematic. An inadequate reduction as assessed on both modalities was only predictive for THA conversion to a certain extent (31–35%). Apparently, many patients are able to function reasonably well despite an inadequate acetabular reduction, perhaps by taking pain medication or by adaptation in terms of avoiding certain activities or lowering their activity level. Specifically in (less active or low-demand) elderly patients, inadequate acetabular reductions have previously shown limited correlation to functional outcome [14]. It is evident that, apart from a poor reduction, many (known and unknown) factors ultimately influence native hip failure and conversion to THA [1–3, 15–18].

While quality of reduction (as evaluated on both post-operative CT as well as PXR) showed an association with native hip survivorship, only an inadequate reduction as assessed on CT was identified as an independent risk factor for conversion to THA. After adjusting for (stronger) confounders, reduction quality based on PXR assessment was no longer found to be a significant risk factor in our patient cohort.

Several studies have sought to identify predictors for clinical or functional outcomes following acetabular fracture surgery [4, 6, 7, 19]. However, a few studies have specifically examined the independent association between quality of reduction and native hip survivorship, and results have been inconclusive [1–3]. In the largest series of 810 patients, a non-anatomical (inadequate) reduction was found to be an independent risk factor for early conversion to THA (within 2 years); [3]. In a study of 285 patients examining long-term hip survivorship, reduction quality was not found to be an independent predictor in older and younger subgroups but only reached significance when measured as step deformity (> 2 mm) on the obturator oblique radiograph in the total cohort [1]. In a further recent study with long-term follow-up, accuracy of reduction was not found to be a risk factor for a composite outcome (including conversion to THA) in acetabular fracture patients who underwent surgical hip dislocation [2]. Importantly, these prior studies all based their assessment of reduction quality on post-operative PXR rather than CT imaging.

Based on our findings, we propose that postoperative CT-based assessment of reduction quality could play a valuable role (particularly) in acetabular fracture research both for reliable evaluation of direct postoperative results as well as for prognostication purposes, for instance following the introduction of novel approaches or surgical implants. In addition, this modality may provide important information to improve the individual surgeon’s technique. In our personal experience and in that of others, CT imaging following acetabular fracture surgery rarely results in re-intervention for misplaced hardware or malreduction [20]. However, in the future, the introduction of intra-operative CT scanning may allow direct revisions based on CT findings. Ultimately, benefits of postoperative CT assessment should be weighed against its apparent drawbacks in terms of radiation risk and increased costs.

Limitations of this study include the large proportion of patients excluded based on the absence of a complete set of postoperative imaging or an inadequate follow-up duration. Nevertheless, it should be considered that this study was designed to directly compare the performance of two methods within the same cohort of patients. In terms of external validity, it appears that our final patient cohort is largely comparable to earlier series of operatively treated acetabular fracture patients. Gender and fracture types were similar [1, 3, 4, 8], but overall conversion rate to THA (24%) was somewhat higher than reported in earlier long-term follow-up studies (14–16%) [1, 3, 4], in part related to our mean patient age (51 years), which was at the higher end of the spectrum as compared to prior study cohorts (ranging from 36–53 years) [1, 4, 20, 21].

## Conclusion

Following acetabular fracture surgery, native hip survivorship is better predicted using CT imaging as compared to PXR assessment. Predicting hip failure and need for THA in patients with inadequate reductions based on both assessment methods remain challenging. While both PXR and CT-based methods are associated with hip survivorship, only an inadequate reduction according to CT assessment was an independent risk factor for conversion to THA. As such CT could present a valuable tool particularly in future research.
